# Effects of *in vitro* selenium addition to the semen extender on the spermatozoa characteristics before and after freezing in water buffaloes (*Bubalus bubalis*)

**Published:** 2012

**Authors:** Kamran Dorostkar, Sayed Mortaza Alavi-Shoushtari, Aram Mokarizadeh

**Affiliations:** 1 *Department of Clinical Sciences, Faculty of Veterinary Medicine, Urmia University, Urmia, Iran; *; 2 *Department of Microbiology, Faculty of Veterinary Medicine, Urmia University, Urmia, Iran.*

**Keywords:** Buffalo, Semen, Selenium supplementation

## Abstract

The aim of the present study was to investigate the effect of *in vitro *supplementation of selenium on fresh and frozen spermatozoa quality of buffalo (*Bubalus bubalis*) bulls. Five healthy buffalo bulls (5 ejaculates from each bull) were used. Each ejaculate was diluted at 37 ˚C with tris-based extender containing 0 (control), 0.5, 1, 2, 4 and 8 µg mL^-1^ sodium selenite and the sperm motility and viability were evaluated at 0 (T_0_) (immediately after dilution), 60 (T_1_) and 120 (T_2_) min after diluting semen. In the second step, semen samples were diluted with tris-egg yolk-glycerol extender containing the same amounts of sodium selenite, cooled to 4 ˚C, equilibrated and semen parameters (motility, viability, membrane integrity and DNA damage) were estimated. Then, the semen was packed in 0.5 mL French straws and frozen in liquid nitrogen. Later, the semen was thawed and analyzed for the same parameters, as well as total antioxidant capacity. Results showed that addition of 1 and 2 µgmL^-1^ selenium to the semen extender significantly increased the sperm motility of fresh and equilibrated semen compared to the control without affecting other parameters. However, in frozen-thawed semen, extenders containing 1 and 2 µg mL^-1^ selenium significantly improved sperm motility, viability, membrane integrity and semen total antioxidant capacity and also resulted in lower DNA damaged sperms. In this study selenium supplementation of semen extender of 4 and 8 µg mL^-1^ had deleterious effects on sperm parameters as early as the samples were prepared for freezing.

## Introduction

Artificial insemination is a valuable tool in genetic improvement programs and a widely used breeding technique in farm animals, especially buffaloes.^1^ However, Semen processing and cryopreservation cause considerable damage to the sperm DNA, motility apparatus, plasma membrane and acrosomal cap, leakage of intracellular enzymes and thus, reduced fertility.^2^^-^^5^ It is well known that oxidative stress plays a major role in the sperm malfunctions via induction of lipid peroxidation to bio-membranes.^6^ Semen contains a variety of antioxidants acting as free radical scavengers to protect spermatozoa against reactive oxygen species (ROS).^7^ Semen processing and cryopreservation decreases the antioxidant defense capacity of semen; the addition of antioxidant in the freezing diluent exerted a protective effect against lipid peroxidation, thereby preserving the metabolic activity and cellular viability.^8^^,^^9^

Selenium (Se) is an essential trace nutrient for humans and animals. Selenium deficiency has been linked to reproductive problems and reduced sperm quality in rats, mice, chickens, pigs, sheep, and cattle,^10^^,^^11^ and dietary Se supplementation has been reported to improve reproductive performance in mice, sheep and cattle;^12^^-^^14^ but high Se intake has been associated with an impaired semen quality.^10^

Selenium is an integral part of glutathione peroxidase (GSH-PX), an enzyme which protects cell internal structures against free radicals and is an antioxidant for cellular membrane lipids.^15^^,^^16^ Glutathione peroxidase activity has been reported in the semen of several species including ram, dog, human, goat, chicken and the bull.^17^^-^20

The literature on the *in vitro *effect of Se on buffalo semen is poor, therefore, this experimental study was conducted to determine the effects of *in vitro* supplementation of different concentrations of sodium selenite (Na_2_SeO_3_) on sperm parameters of fresh and frozen semen with the aim of finding a procedure to obtain an improved semen quality after freeze-thawing.

## Materials and Methods

**Semen collection and processing. **Five healthy buffalo bulls (3-5 years age) kept at Buffalo Breeding Center of Northwest of Iran, Urmia (37˚ 33΄ N, 45˚ 4΄ E) under common management conditions were used. Semen samples were collected using an artificial vagina; only samples of at least 80% initial progressive motility were used. A total number of 25 semen samples (5 ejaculates from each bull) were used in the study. Each ejaculate was split into 6 portions and diluted at 37 ˚C with tris-based extender (tris 2.660 g, glucose 1.200 g, citric acid 1.390 g, cysteine 0.139 g, distilled water up to 100 mL) containing 0 (control), 0.5, 1, 2, 4 and 8 µg mL^-1^ sodium selenite (Na_2_SeO_3_, Sigma Chemical Co., St. Louis, MO, USA) and the sperm motility and viability were assessed at 0 (T_0_) (immediately after dilution), 60 (T_1_) and 120 (T_2_) min after diluting semen. In the second step, a tris-egg yolk-glycerol extender containing the same amounts of sodium selenite was prepared, semen samples (6 portions per ejaculate) diluted and left for 2 hr to cool to 4 ˚C and equilibrate in an equilibration chamber of 4 ˚C for 4 hr and, semen parameters (progressive motility, viability, membrane integrity and DNA damage) were estimated. Then, the semen was packed in 0.5 mL French straws, frozen in liquid nitrogen according to the procedure reported by Sukhato *et al*., and stored until analysis.^21^ Later, the frozen semen was thawed in 37 ˚C water bath for 30 sec. The same parameters and total antioxidant capacity (TAC) of the frozen-thawed semen were estimated.

**Semen quality assessment. **The following assays were conducted on fresh, cooled and frozen-thawed buffalo semen.

**Sperm progressive motility and viability. **Sperm motility was estimated using a computer assisted system of analysis (CASA) (Hoshmand Fannavar [HFT] CASA, Version 6, Amirkabir Medical Engineering Co., Tehran, Iran). Sperm viability was evaluated using Eosin-Nigrosin staining method.^22^

**Sperm plasma membrane integrity. **Sperm membrane integrity assessed before and after freezing by the hypo-osmotic swelling test (HOST), as described by Jeyendran *et al*.^23^ In brief, the hypo-osmotic solution (osmotic pressure ≡150 mOsmol kg^-1^, Osmomat 030; Gonotec, Berlin, Germany) was prepared by dissolving 0.73 g sodium citrate and 1.35 g fructose in 100 mL of distilled water. Hypo-osmotic solution (500 µL) was mixed with 50 µL of semen and incubated at 37 ˚C for 40 min. After incubation, a drop of semen sample was examined under a phase-contrast microscope (Model BX41, Olympus Corp., Tokyo, Japan) (400×) and 200 spermatozoa were counted in at least five different fields for their swelling characterized by coiled tail indicating intact plasma membrane.

**DNA Damaged Sperms. **DNA damage was detected using acridine orange staining technique, according to the method of Katayose *et al*.^24^ Briefly, medium-thick smears of sperm on the glass slides were air dried, fixed for 2 hr in freshly prepared Carnoy’s solution (Methanol/ Glacial acetic acid), air dried again, and stained with acidic work solution containing Acridine Orange hemi (zinc chloride) salt (Sigma Chemical Co., St. Louis, MO, USA). All slides were examined on a fluorescence microscope (Model GS7, Nikon, Tokyo, Japan). A total number of 200 cells were counted on each slide and classified as green or red based on differences in their fluorescent color.

**Total antioxidant capacity.** Total antioxidant capacity of the frozen-thawed semen was estimated by a commercial kit (Antioxidant Capacity Assay Kit, Cayman Chemical Co., Ann Arbor, USA) and spectrophotometer (Model No. 330, Camspec Ltd., Cambridge, UK) at 520 nm wavelengths. 

**Statistical analysis. **The obtained data was analyzed using SPSS software (version 18 for windows, SPSS Inc., Chicago, IL, USA). Results were quoted as the mean ± standard error of mean (SEM) and significance was set at *p* < 0.05. One-way analysis of variance (ANOVA) was used to compare the means. Duncan's test was used for the multiple comparisons of all groups.

## Results

**Fresh semen. **Adding 1 and 2 µg mL^-1^ Se to the extender significantly increased the motility of the sperm in fresh semen at T_1_ and T_2_ compared to the control (85.7± 0.9 [1 µg mL^-1^] and 86.9 ± 0.8% [2 µg mL^-1^] vs. 80.5 ± 1.2 % [control] at T_1_; 81.1 ± 1.8 [1 µg mL^-1^] and 83.2 ± 1.2 % [2 µg mL^-1^] vs. 76.3 ± 1.4 % [control] at T_2_, respectively), the results of 2 µg mL^-1^ at T_1_ and T_2 _were better than other concentrations (Table 1). But, the sperm viability was not significantly affected by addition of Se (Table 1), while 8 µg mL^-1 ^Se had significantly adverse effects on sperm motility and viability at all the measurements (T_0_, T_1_ and T_2_). 

**Diluted equilibrated semen. **In equilibrated semen addition of 2 µg mL^-1^ Se increased sperm motility significantly compared to the control (77.4 ± 1.7 % vs. 70.1 ± 1.6 %) and did not affect viability and plasma membrane integrity of the spermatozoa while, these parameters were significantly decreased by addition of 4 µg mL^-1^ Se (Table 2). Only addition of 8 µg mL^-1^ Se significantly decreased motility viability and plasma membrane integrity of the spermatozoa and increased DNA damaged spermatozoa before freezing the semen. 

**Frozen-thawed semen. **In frozen-thawed semen, addition of 1 and 2 µg mL^-1^ Se preserved motility (46.6± 1.9 [1 µg mL^-1^] and 47.5 ± 1.8% [2 µg mL^-1^] vs. 40.3 ± 1.6%[control]), viability (64.8 ± 1.5 [1 µg mL^-1^] and 67.4 ± 1.9% [2 µg mL^-1^] vs. 59.1 ± 1.5% [control]) and membrane integrity (63.4 ± 1.3% [1 µg mL^-1^] and 66.1 ± 1.7% [2 µg mL^-1^] vs. 56.5 ± 1.65% [control]) compared to the control (Table 3). Selenium at concentrations of 4 and 8 µg mL^-1 ^had a deleterious effect on these parameters. Compared to the control, addition of 2 µg mL^-1^ Se resulted in lower DNA damaged sperms (9.0 ± 0.35% vs. 10.8 ± 0.36%) but 8 µg mL^-1^ Se increased DNA damage rate significantly (13.1±0.40% vs. 10.8 ± 0.36%). Total antioxidant capacity (TAC) of the frozen thawed semen was increased by adding 2 µg mL^-1^ Se (from 62 ± 3.5 to 78 ± 3.5 µmol L^-1^) while it was suppressed by addition of 8 µg mL^-1^ Se (42± 3.9 µmol L^-1^) (Table 3). 

**Table 1:** Effects of different Se concentrations on motility and viability (Mean ± SEM) of spermatozoa in fresh semen (n = 25).

**Parameters**			**Concentrations (µg mL** ^-1^ **)**				
	**Time**	**Control**	**0.5**	**1**	**2**	**4**	**8**
**Motility**	**T**_0_	83.8 ± 1.0^a^	83.3 ± 1.0^a^	84.1 ± 1.1^a^	83.8 ± 1.0^a^	81.4 ± 1.2^ab^	79.6 ± 1.6^b^
	**T**_1_	80.5 ± 1.2^a^	81.6 ± 1.1^a^	85.7 ± 0.9^b^	86.9 ± 0.8^b^	75.6 ± 1.5^c^	72.4 ± 1.8^c^
	**T**_2_	76.3 ± 1.4^a^	78.4 ± 1.3^ab^	81.1 ± 1.8^cb^	83.2 ± 1.2^c^	70.8 ± 1.5^d^	61.3 ± 1.6^e^
**Viability**	**T**_0_	87.1 ± 1.2^a^	86.8 ± 1.0^a^	87.5 ± 0.8^a^	88.8 ± 1.2^a^	86.4 ± 1.1^a^	83.7 ± 1.3^b^
	**T**_1_	85.6 ± 1.2^a^	85.1 ± 1.2^a^	86.4 ± 1.0^a^	86.2 ± 1.1^a^	83.2 ± 1.3^ab^	80.5 ± 1.6^b^
	**T**_2_	81.5 ± 1.7^a^	81.2 ± 1.8^a^	82.7 ± 1.6^a^	83.5 ± 1.5^a^	80.1 ± 1.4^a^	74.7 ± 1.7^b^

a, b, c Superscript letters indicate significant differences within rows (*p* < 0.05); T_0_ = 0, T_1_ = 60, T_2_ = 120 min after dilution.

**Table 2:** Effect of different Se concentrations on sperm parameters (Mean ± SEM) after equilibrium time (n = 25).

**Parameters**			**Concentrations (µg mL** ^-1^ **)**				
		**Control**	**0.5**	**1**	**2**	**4**	**8**
**Motility (%)**		70.1 ± 1.6^a^	71.0 ± 1.7^ab^	76.3 ± 1.8^bc^	77.4 ± 1.7^c^	64.3 ± 1.2^d^	56.9 ± 1.3^e^
**Viability (%)**		77.2 ± 1.5^a^	78.0 ± 1.7^a^	78.5 ± 1.8^a^	79.6 ± 1.8^a^	74.9 ± 1.6^a^	69.6 ± 1.9^b^
**Membrane Integrity (%)**		78.6 ± 1.2^a^	79.7 ± 1.1^a^	81.6 ± 1.3^a^	81.1 ± 1.1^a^	74.5 ± 1.5^b^	68.5 ± 1.7^c^
**Damaged DNA (%)**		3.0 ± 0.25^a^	2.7 ± 0.23^a^	3.0 ± 0.24^a^	2.4 ± 0.22^a^	3.0 ± 0.24^a^	4.1 ± 0.26^b^

a, b, c Superscript letters indicate significant differences within rows (*p* < 0.05).

**Table 3:** Effect of different Se concentrations on frozen sperm parameters (Mean ± SEM) after thawing (n = 25).

**Parameters**			**Concentrations (µg mL** ^-1^ **)**				
		**Control**	**0.5**	**1**	**2**	**4**	**8**
**Motility (%)**		40.3 ± 1.6^a^	43.1 ± 1.8^ab^	46.6 ± 1.9^b^	47.5 ± 1.8^b^	32.5 ± 1.7^c^	26.7 ± 1.9^d^
**Viability (%)**		59.1 ± 1.5^a^	61.3 ± 1.7^ab^	64.8 ± 1.5^bc^	67.4 ± 1.9^c^	53.6 ± 1.9^d^	41.6 ± 1.8^e^
**Membrane Integrity (%)**		56.5 ± 1.6^a^	59.1 ± 1.8^ab^	63.4 ± 1.3^bc^	66.1 ± 1.7^c^	48.4 ± 1.8^d^	38.5 ± 1.9^e^
**Damaged DNA (%)**		10.8 ± 0.36^ab^	10.1 ± 0.27^ac^	9.5 ± 0.36^cd^	9.0 ± 0.35^d^	11.7 ± 0.37^b^	13.1 ± 0.40^e^
**TAC (µmol L** ^-1^ **)**		62.0 ± 3.5^a^	64.0 ± 3.9^a^	64.0 ± 3.9^a^	78.0 ± 3.5^b^	58.0 ± 3.6^a^	42.0 ± 3.9^c^

a, b, c Superscript letters indicate significant differences within rows (*p* < 0.05).

## Discussion

It is well known that the main steps of cryopreservation, such as cooling and freeze-thawing carries both physical and chemical stress on sperm membranes,^25^ but also an oxidative stress^26^ resulting in impaired physiological sperm characteristics. Several studies have tested effects of adding antioxidant additives in semen extenders on the protection of spermatozoa during semen processing and cryopreservation.^27^^,^^28^ There are studies that reported on a significant positive correlation between Se levels in seminal plasma and sperm quality.^29^^,^^30^ There is little information available on *in vitro* effects of Se on buffalo semen, thus, this study was designed to investigate the effects of different amounts of Se supplementation of the extender and examine its *in vitro* effects in an attempt to find a way for preserving the quality of the buffalo sperms through freezing. At first, fresh semen was supplemented by different doses of selenium at different times to investigate the dose and time effect on sperm motility and viability. Then, some other sperm parameters, such as plasma membrane integrity, DNA damage and total antioxidant capacity of the semen were included to evaluate the degree of possible damages in equilibrated and frozen- thawed semen. With the view that fresh semen has intact sperms and some parameters, such as total antioxidant capacity of the seminal plasma, had been previously assayed in fresh semen^31^ these parameters were not estimated in fresh semen.

Our results showed that *in vitro *Se supplementation at 2 µg mL^-1 ^doses can improve the quality of fresh and frozen semen of buffalo bulls as compared to the non-supplemented control group. The present results are in agreement with other *in vitro* and *in vivo* studies in mice, human, ram, bovine and other spieces.^11^^,^^32^^-^^38^

The total antioxidant capacity of seminal plasma represents the sum of the potential anti-ROS enzymes, such as GSH-PX.^39^ Selenium is a component of the enzyme GSH-PX that protects cellular membranes and lipid containing organelles from per-oxidative damage.^40^ Zhang *et al.* reported that in the cell culture, selenium in the form of selenite helps the cells to detoxify the medium to protect them from oxidative damages.^41^ Addition of selenite to semen extender in this study increased total antioxidant capacity, in a dose dependent manner. The present results are in agreement with the findings of earlier researchers who reported that Se supplementation led to significant increases in the mean seminal plasma Se activity.^33^^-^^42^ Furthermore, Contri *et al*. reported a positive correlation between sperm parameters and total antioxidant capacity in seminal plasma.^43^ Protective effect of Se supple-mentation on frozen-thawed sperm viability and membrane integrity and also sperm motility before and after freezing in the test group of 1 and 2 µg mL^-1^ Se observed in this study may be explained by the amplified antioxidant enzymes suggesting that Se supplementation could increase the ability of seminal plasma to reduce the oxidative stress. In this study a significant decrease in post-thaw DNA damage levels were observed in Se supplemented groups as compared to the control that was most probably due to increased antioxidant capacity. Enhancement of sperm motility in fresh, equilibrated and frozen semen observed in this study may be due to changes in metabolism of sperm and increased oxygen consumption as reported by Pratt *et al*. and Marin-Guzman *et al*., who showed that Se supplementation enhances enzymatic rates of ATP-utilizing and ATP-regenerating pathways of the sperm, which are assessed by motility and oxygen consumption of the sperm.^36^^,^^44^ On the other hand, Se has been shown to modulate adenylate cyclase and cyclic adenosine-mono-phosphate related signaling events, as well as protein kinases.^44^

Our results revealed that addition of sodium selenite to the semen extender, in concentrations of 1 and 2 µg mL^-1^ significantly stimulated and improved motility of fresh and equilibrated sperm, without affecting viability. These results suggested that Se promoted motility in sperm which would otherwise be non-motile, but were still viable and also, indicated that Se at this level before freezing did not affect any processes which were detrimental to the sperm.^36^^,^^46^ These results were in accordance with the findings of earlier researchers in the ram and bovine sperm.^34^^-^^36^^,^^47^ However, in frozen-thawed semen Se additive of 1 and 2 µg mL^-1^ had a positive impact on both motility and viability. It may be due to the fact that during storage, the susceptibility of spermatozoa to the oxidative stress is significantly increased, probably due to initiation of spontaneous lipid peroxidation;^9^ in this way, the protective effect of Se enrichment of extender has been clearly demonstrated.

In this study supplementation of semen extender with high levels of Se (8 µg mL^-1^) had deleterious effects on sperm parameters as early as the sample preparation phase for freezing. This is in agreement with the findings of earlier reports on the human, rat, ram and bovine; ^34^^-^^36^^,^^48^^,^^49^ that suggested sperm function can be impaired by excess Se as well as by Se deficiency. Toxic effect of Se might be attributed to destructive impact of high microelement concentration on the spermatozoa insert, regarded as an energetic area of male gamete and with the impairment of processes, including physiological oxidation or cell respiration, occurring in mitochondria.^48^ Studies have shown that Se is localized in the keratinous outer membrane of sperm mitochondria implies that Se plays the main role in maintaining a proper composition of this structure.^50^ Although this study indicated that supplementation of the semen extender with Se may beneficially affect the quality of the semen; it seems that the effect of Se can be different, depending on doses, time lapse and phase of processing. 

In conclusion, this study indicates that Se supplementation may help to ameliorate negative effects of water buffaloes semen storage on characteristics of sperm quality; improved sperm motility, viability, plasma membrane integrity and DNA damage, with higher values obtained after adding 2 µg mL^-1^ sodium selenite. Selenium exerts its effects in a dose-dependent manner and at high levels of 4 and 8 µg mL^-1^ is deleterious for the sperm.
